# A variant of uncertain significance in COL7A1 in Bart syndrome: A case report

**DOI:** 10.1016/j.jdcr.2026.06.062

**Published:** 2026-07-03

**Authors:** Tatum La, Katherine Gordon

**Affiliations:** a Medical School, University of Texas Southwestern Medical Center, Dallas, Texas; b Department of Dermatology, University of Texas Southwestern Medical Center, Dallas, Texas

**Keywords:** aplasia cutis congenita, Bart syndrome, collagen type VII, epidermolysis bullosa

## Introduction

Bart syndrome, also known as aplasia cutis congenita type VI, is a condition characterized by epidermolysis bullosa, congenital absence of the skin, and nail abnormalities.^1^ Here, we present a case of Bart syndrome with a unique presentation and a variant of uncertain significance.

## Case Report

**Fig 1:**
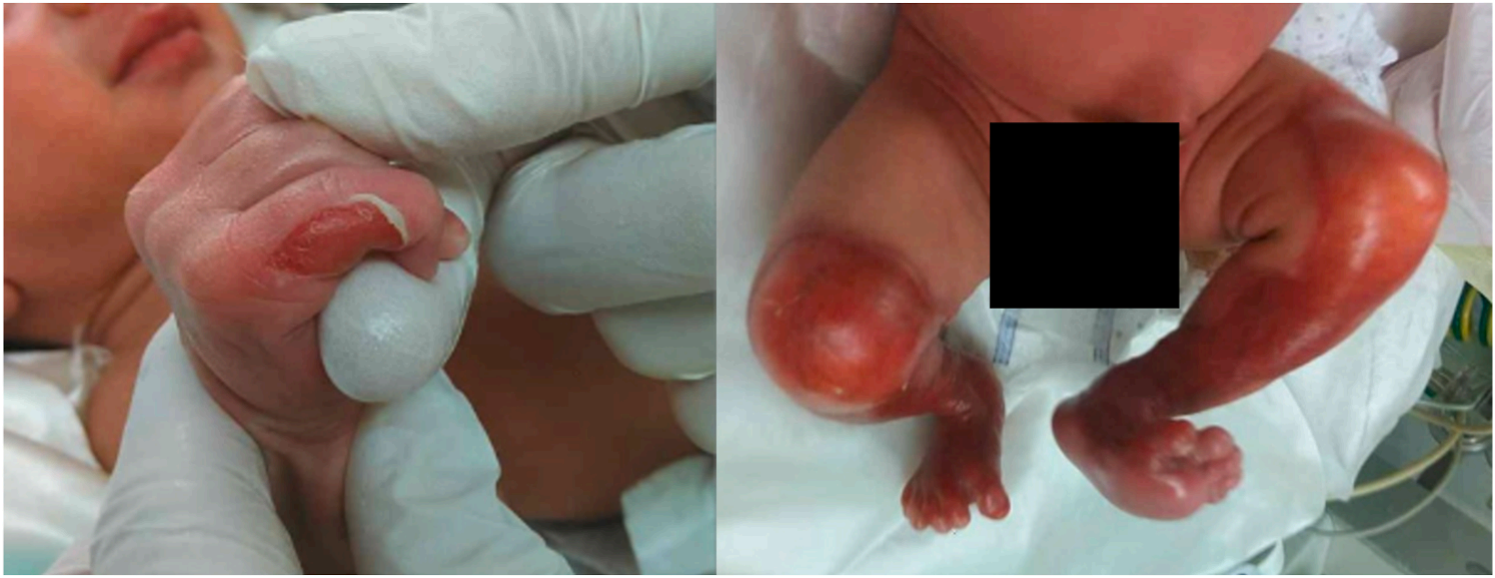
Right hand and bilateral lower extremities at 3 days old.

A male infant was born at 37 4/7 weeks gestation via vaginal delivery to a mother with suspected chorioamnionitis. He was admitted to the neonatal intensive care unit given extensive congenital absence of skin noted at birth. On physical examination, skin was absent on bilateral legs from the knees to the feet, including the soles of the feet and the great toe (Fig 1). His right thumb nail bed was ecchymotic, and ruptured vesicles and bullae were noted on the bilateral hands and left wrist. Mucosal erosions were noted on his upper and lower gingiva. Wound culture from his leg wounds revealed methicillin-resistant Staphylococcus aureus, and he was treated with vancomycin and clindamycin.

He was placed in a humidified isolette and received wound care with Aquaphor, Xeroform wrap, and gauze. He received alternating morphine and acetaminophen in addition to gabapentin for pain management.

Invitae Epidermolysis Bullosa and Palmoplantar Keratoderma Panel identified 2 COL7A1 mutations: c.6769G>A (p.Gly2257Arg), heterozygous, likely pathogenic and c.5338C>T (p.Arg1780Trp), heterozygous, uncertain significance. In addition, there was DSG1 c.2956G>A (p.Ala986Thr) mutation that was heterozygous and of uncertain significance. Based on the COL7A1 c.6769G>A (p.Gly2257Arg mutation, he was diagnosed with dystrophic epidermolysis bullosa. Mode of inheritance (autosomal recessive vs dominant) is yet to be elucidated. The infant was discharged, and his family was given instructions for continued wound care and pain management.

At 28 days old, he was re-admitted due to increased malodorous drainage from his lower extremity wounds. Aerobic cultures showed moderate growth of methicillin-resistant Staphylococcus aureus and Escherichia coli, which was treated with IV clindamycin and trimethoprim-sulfamethoxazole. One week later, he was seen for outpatient follow-up in the dermatology clinic, and his infection was noted to be resolving. On physical exam, there were eroded, atrophic erythematous patches with granulation tissue on the bilateral hands, lower extremities, and feet. One thumbnail was dystrophic, and anonychia was noted on all toes. There was a shallow erosion on the lower mucocutaneous lip and lower gingiva. Scattered excoriations were noted on his trunk.

Due to lower limb deformities, he was referred to pediatric orthopedics. He was diagnosed with calcaneovalgus and absence of the great toes bilaterally, but his range of motion at the ankles and knees was preserved. Physical therapy was initiated. The infant also saw pediatric ophthalmology who noted corneal haze and scarring as a potential complication of epidermolysis bullosa.

**Fig 2:**
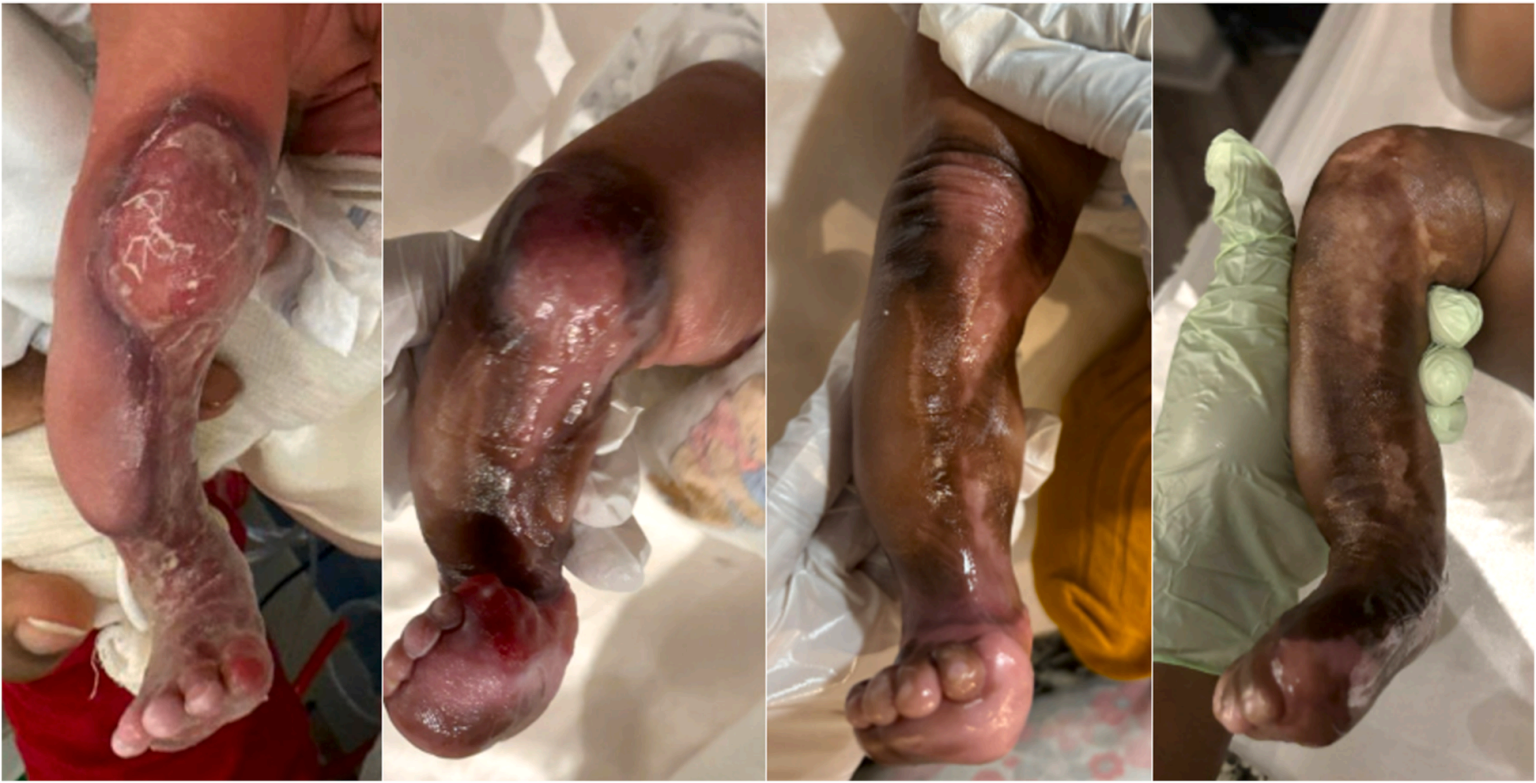
Right lower extremity at 9 days old, 38 days old, 51 days old, and 145 days old (left to right, respectively).

**Fig 3:**
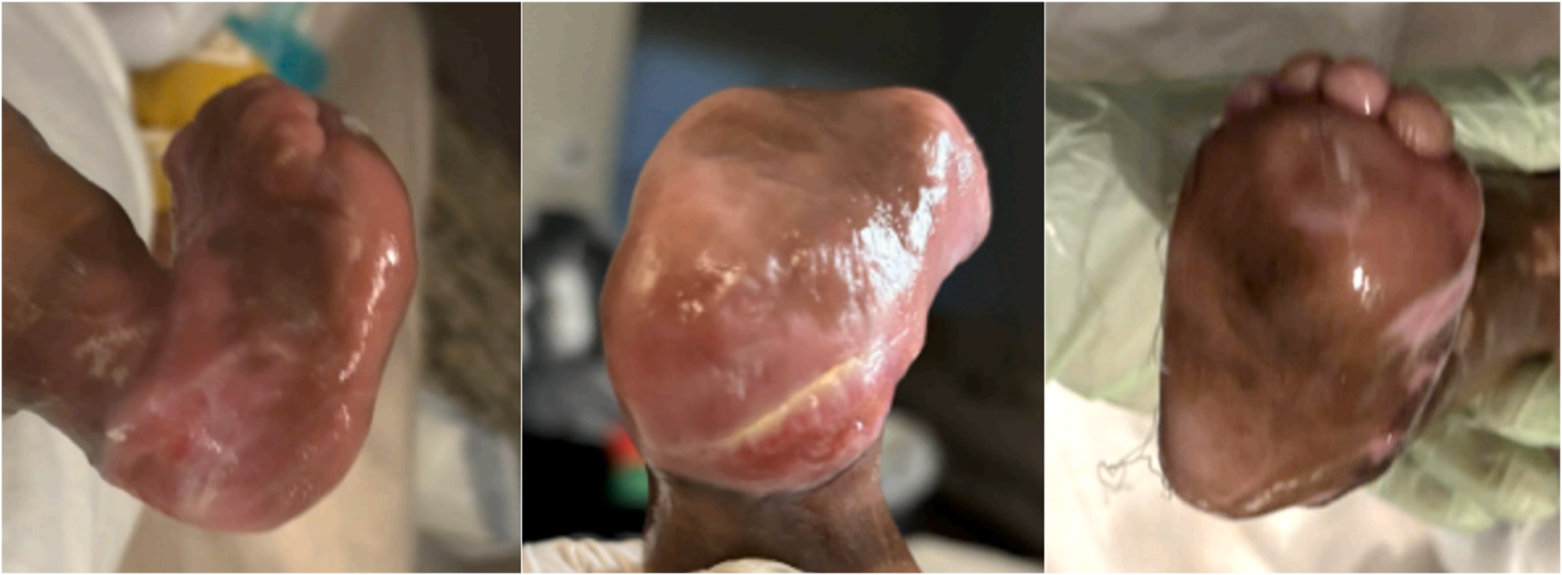
Right foot at 38 days old, 51 days old, and 145 days old (left to right, respectively).

**Fig 4:**
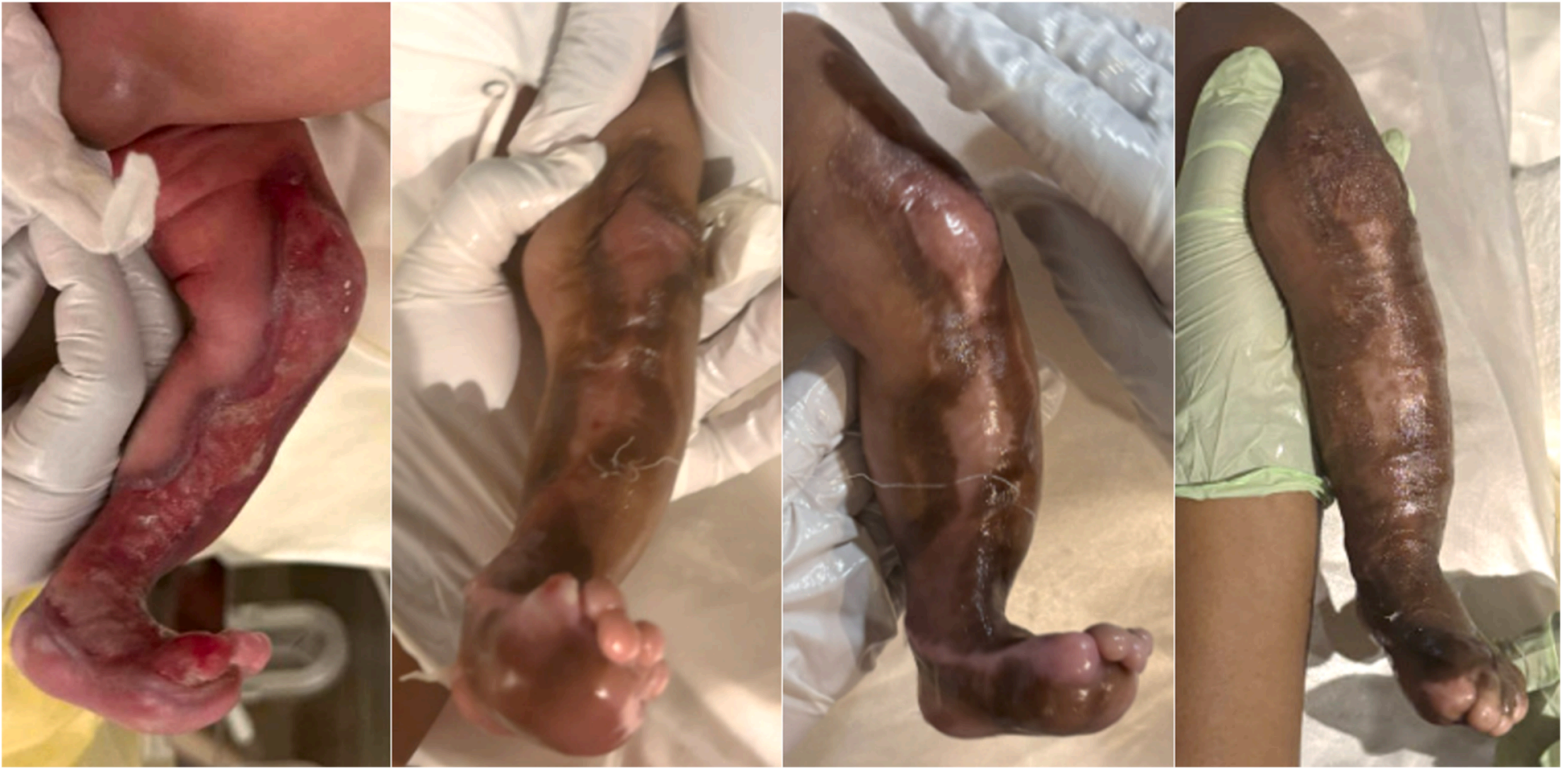
Left lower extremity at 9 days old, 38 days old, 51 days old, and 145 days old (left to right, respectively).

Over time, much of the patient’s initial erosions healed, with resultant dyspigmented patches scattered on the upper extremities and healed atrophic patches on the bilateral lower extremities. (Fig 2, Fig 3, Fig 4). He continues to have blistering on his oral mucosa, infrequent blisters on his upper and lower extremities, scattered milia, and anonychia of all toes.

## Discussion

Aplasia cutis congenita (ACC) is a heterogenous group of disorders characterized by areas of absent skin at birth. Under the Frieden classification, there are 9 distinct groups of ACC, with Group 6 being ACC with epidermolysis bullosa. Bart syndrome is a variant of ACC that was first described in 1966 as a triad of congenital absence of skin affecting the lower extremities, blistering of skin and mucous membranes, and congenital absence and deformity of nails.^1^ Our patient’s presentation aligns with the traditional picture of Bart Syndrome, and his case is especially notable due to the severity of presentation and 2 genetic variants of uncertain significance, COL7A1 c.5338C>T (p.Arg1780Trp) and DSG1 c.2956G>A (p.Ala986Thr). While mutations in DSG1 are not associated with epidermolysis bullosa or Bart syndrome, COL7A1 mutations are classically implicated in dystrophic epidermolysis bullosa.^2^ COL7A1 encodes the anchoring fibrils responsible for dermal-epidermal adhesion, and dysfunctional collagen VII leads to sublamina densa cleavage. Patients with epidermolysis bullosa and congenital absence of skin commonly have glycine or arginine substitutions in the triple-helix domain of collagen VII or near triple-helix domain interruptions.^3^ While the COL7A1 c.5338C>T (p.Arg1780Trp) mutation has not been previously reported, it is within the triple-helix domain and thus aligns with the pathogenesis of dystrophic epidermolysis bullosa.

Further, our patient acquired auto-amputation of the great toes bilaterally during infancy, a finding that has been documented in epidermolysis bullosa but not specifically in Bart syndrome.^4^ Few cases of dystrophic epidermolysis bullosa with foot deformities have been described, but these cases involve brachydactyly of the great toes rather than auto-amputation.^5^

Multidisciplinary treatment is indicated for Bart syndrome. For dermatologic management, gentle skin care that minimizes friction and trauma to the skin and proactive wound care are imperative. Additional recommendations may include silk sheets and decreased frequency of bathing due to burdens of wound dressing changes. Given the widespread effects of epidermolysis bullosa on skin and mucosal surfaces, additional multidisciplinary care includes other pediatric subspecialties such as orthopedics, otolaryngology, ophthalmology, pain management, gastroenterology, and nutrition. For congenital absence of skin associated with Bart syndrome, treatment varies based on the size and location of the lesion. Small and superficial areas often heal with local wound care, while more extensive lesions on areas such as the scalp may require surgical management.^6^

Bart syndrome is a rare condition that requires prompt diagnosis, vigorous wound care, and thorough counseling for families of children with the condition. A combination of multidisciplinary clinical teams, patient education, and proactive wound care ensures that patients with Bart syndrome have the best possible prognosis.

## Conflicts of interest

None disclosed.
